# Information fusion-based approach for studying influence on Twitter using belief theory

**DOI:** 10.1186/s40649-016-0030-2

**Published:** 2016-09-22

**Authors:** Lobna Azaza, Sergey Kirgizov, Marinette Savonnet, Éric Leclercq, Nicolas Gastineau, Rim Faiz

**Affiliations:** 1 LE2I Laboratory-UMR6306-CNRS-ENSAM, University of Burgundy Franche-Comté, 9 Avenue Alain Savary, 21078 Dijon, France; 2 LARODEC, IHEC, University of Carthage, Tunis, Tunisia

**Keywords:** Social influence, Information fusion, Multi-level fusion, Belief theory, Twitter network

## Abstract

Influence in *Twitter* has become recently a hot research topic, since this micro-blogging service is widely used to share and disseminate information. Some users are more able than others to influence and persuade peers. Thus, studying most influential users leads to reach a large-scale information diffusion area, something very useful in marketing or political campaigns. In this study, we propose a new approach for multi-level influence assessment on multi-relational networks, such as *Twitter*. We define a social graph to model the relationships between users as a multiplex graph where users are represented by nodes, and links model the different relations between them (e.g., *retweets*, *mentions*, and *replies*). We explore how relations between nodes in this graph could reveal about the influence degree and propose a generic computational model to assess influence degree of a certain node. This is based on the conjunctive combination rule from the belief functions theory to combine different types of relations. We experiment the proposed method on a large amount of data gathered from *Twitter* during the European Elections 2014 and deduce top influential candidates. The results show that our model is flexible enough to to consider multiple interactions combination according to social scientists needs or requirements and that the numerical results of the belief theory are accurate. We also evaluate the approach over the CLEF RepLab 2014 data set and show that our approach leads to quite interesting results.

## Background

Nowadays, online social networks, such as *Twitter*, gather people together and empower their relationships with new forms of cooperation and communication. As a result of its massive popularity, *Twitter* is exploited as a platform for very different purposes, such as marketing or political campaigns [1]. One of the most distinctive characteristics of *Twitter* is the information diffusion through social links. In fact, links between users impact the information flow and thus indicate the user’s influence on others. Some users, called influentials, are more able than others to diffuse information to a huge number of users. Therefore, determining influential users in a network is a secret key of success for achieving a large-scale information diffusion at low cost.

The Merriam-Webster dictionary has defined influence as “The power or capacity of causing an effect in indirect or intangible ways.” Despite the large number of influence theories in sociology, there is no obvious way to measure such a power. Focusing on an individual’s potential to engage others in a certain act, Alex et al. [2] have defined influence on *Twitter* as the potential of a user’s action to initiate a further action by another user. The term “action” means the different possible relationships between users. Hence, measuring influence on *Twitter* is not that simple as *Twitter* provides several forms of relations. A user can *follow* another one, which allows him to see *tweets* and information about the user he *follows*. He is also able to *retweet* a *tweet*, this exposes the *tweet* to his *followers* who can also *retweet* it. A user can *mention* another one using the “@” prefix if he wants to address or to show him the *tweet*. Besides, a user can *reply* to another’s *tweet* and thus creates a conversation with him. Moreover, a user can *like* another *tweet* using the small heart icon under the *tweet* to express his appreciation to its content. And finally, interactions can be created from the use of different relations in sequence such as a *retweet* containing a *mention*. These different relations are what made *Twitter* a multi-relational network [3, 4] on which possible links can *retweet*, *mention*, *reply*, *follow* or *like*. While measuring influence, the choice of these relations depends on understanding the subject and domain area [5].

Influence assessment poses three main challenges. The first is the diversity of relations and interactions on which we can rely to compute influence. Moreover, joint use of relations in a *tweet* can have different meanings. For example, using several *mentions* of media at the end of a *tweet* allows to expose the *tweet* in the largest number of users. It is important to combine relations to establish a general influence measure that considers the different types of relations and interactions among users. The second challenge is the consideration of indirect influence. In some cases, the influence is not direct, it extends to a user through intermediates users. For example, a user may *retweet* another’s *tweet* indirectly through an intermediate user. It is necessary to measure the influence regarding the direct and indirect interactions in the network. The third challenge is related to uncertainty when the combination of relations and interactions is performed, due to their diversity, it is difficult to assign importance weights to the different relations, and it is even more difficult when they are combined.

In this paper, we extend the model proposed in [6], we focus on extensibility, and we develop an algorithm for multi-level fusion of information about different relations and interactions. Our contributions are manifold. To evaluate influence, we define the influence graph allowing us to capture the relationships between users as a labeled multiplex graph where users are represented by nodes, and links model the different relations between them. After that, we combine the relations obtained from the graph to assess influence. The measure can be established between a couple of users by taking into account different relationships or interactions between them or it may also assess a user’s global influence in the network considering all the relations and interactions where his peers are involved in. We also consider uncertainty in the measurement process. We define a theoretical framework, to compute influence, based on the conjunctive combination rule for belief functions theory and Smets rule [7] to fusion and combine information. The proposed approach is flexible and thus, indirect influence in the graph can also be considered. In this case, the influence assessment considers influence exercised on indirect nodes (e.g. a user may *retweet* another’s *tweet* indirectly through an intermediate user). This spans the influence on multiple levels based on a multi-level influence graph. An evaluation through experiments is proposed. It is based on real data gathered from *Twitter* in the TEE 2014 project during the European Election campaign in 2014. We also conduct experiments on the CLEF RepLab 2014 data set, which contains *Twitter* data including influence-annotated *Twitter* profiles. We take advantage of these manual annotations to analyze our results and study the importance of the belief theory consideration in the influence assessment.

The rest of this paper is organized as follows: "Literature review," "Proposed approach," and "Experiments and results" sections show the review of this study, our proposed approach, and experimental results, respectively. Finally, "Conclusion" section concludes this paper.

## Literature review

In this section, we review studies of influence assessment in *Twitter* and remind the basic concepts of belief functions theory on which our approach is based.

### Influence in *Twitter*

Researchers have been interested in assessing influence in social networks, and many approaches were provided to rank users according to their influence [8]. Some researches are based on **network topology** and centrality measures [9]. Others approaches try to establish a ranking of nodes using **diffusion-based** or random-walk-based algorithms like HITS [10] algorithms or PageRank [11]. A novel family extends network topology approaches to take into account **information fusion** about different interactions that can be considered in the influence assessment. In the following, we present major works on *Twitter* for each type of approach.

While measuring users influence in *Twitter*, many criteria can be considered. Leavitt et al. [2] use four features to measure influence, which are: *replies, retweets,* and *mentions* in addition to number of *followers*. They give statistics related to these measures and do not offer a global influence score based on all the proposed criteria. Cha et al. [12] define three influence measures in *Twitter*, the indegree influence, which is the number of *followers*, indicating the size of a user’s audience or popularity; the *mention* influence corresponds to the number of a user’s *mentions*, indicating his ability to engage others in *mentions*; and the *retweet* influence, which is the number of *retweets*, indicating the ability of a user to write content to be forwarded to others. The authors compute the value of each relation for 6 million users and compare them. To do this, they sort users according to each different relation, after that, they quantify how a user’s rank varies across different relations. Spearman’s rank correlation is used as a measure of the association strength between two rank sets. They found that *followers* number represents a user’s popularity, but is not related to other important relations, such as *retweets* and *mentions*. Their result suggests that *followers* number alone reveals very little about a user’s influence. This research does not provide a global influence measure and only influence measures according to each relation separately. Chen et al. [13] propose a local ranking method named ClusterRank, which considers the number of neighbors and the clustering coefficient. Bakshy et al. [14] followed a different approach to estimate influential users: they use shortned URL diffusion cascades and consider that users producing the largest cascades are the most influential. The presented results are obtained from a survey of 1.6 million users over a period of two months in 2009. In this work, the definition of influence is limited to the ability to be the first to publish URL which is then *retweeted* by followers. Brown et al. in [15] believe that the location of a node in the network may play a more important role than its indegree. For example, a node located in the center of the network, having few highly influential neighbors, may be more influential than a node having a larger number of less influentials neighbors. Considering this fact, *k-shell decomposition* algorithm can be useful [16]. Basically, the principle of the *k-shell decomposition* is to assign a core index *ks* to each node such that nodes with the lowest values are located at the periphery of the network, while nodes with the highest values are located in the center of the network. The innermost nodes thus form the core of the network. They observe that the results of the *k-shell* decomposition on *Twitter* network are highly skewed. Therefore they propose a modified algorithm that uses a logarithmic mapping, to produce fewer and more meaningful *k-shell* values. Correlation between users relations were considered in [17] to identify and measure social influence as a source of correlation between the individuals behaviors with social ties. Authors study the phenomenon that a user’s behavior can induce his/her friends to behave in similar way. To do this, they use logistic regression to quantify social correlation. This is measured as a function of only one variable: the number of active friends the user has. After this, the *shuffle test* is used to decide if influence is a likely source of correlation. The techniques used provide only a qualitative indication of the influence existence and not a quantitative measure. Qasem et al. [18] presented a new approach of influential users detection. The proposed approach detects the users who increase the size of social network by attracting new users into the network. In [19], users review the features that can be extracted from *Twitter* for the purpose of user classification and detecting influential users in real-life based on their *Twitter* profile, they cite many features such as scalar features (e.g., number of followers), users interactions and term occurrences (URLs, punctuation, etc.). After that, the authors use non-linear classifier under the form of kernelized SUM and logistic regression. It consists at representing a user under various forms of bags of words. The results are interesting but are valid only for the considered data set and restricted to the used domains (automotive and banking domains).

The disadvantage of the network topology-based algorithms is to consider information about the users, and not to consider the interaction among users through a sequence of relations. In *Twitter*, the user’s influence is impacted by the information diffusion between the users. Nevertheless, these studies help us to recognize the criteria to take into account in the influence assessment.

Other researches propose to rank nodes using **diffusion-based** or random-walk-based algorithms, with a common assumption that a node is expected to be influential if it points to many highly influential neighbors. In this context, user’s influence were ranked based on the classical random walk algorithm, such as PageRank. The main idea behind PageRank is that “more important pages (web sites) are likely to receive more links from other pages.” Many variants of the PageRank algorithm were proposed to improve it and adapt it to *Twitter*. A notable one was TunkRank [20], and it uses a constant to represent the *retweet* probability, combined with the people whom the user concerned and the fans who concerned this user. The user’s influence was the expected number of the people influenced by the released information in TunkRank. Ghosh et al. [21] propose Collusionrank, a PageRank-like approach, to overcome link farming in *Twitter*. They negatively bias the initial scores towards nodes identified as spammers. Then, since a user should be penalized for following spammers and not for being followed by spammers, the Collusionrank score of a node is computed based on the score of its *followings* (instead of its *followers*). Thus, users who *follow* a larger number of spammers, or who *follow* those who in turn *follow* spammers, get a negative score of higher magnitude and are pushed down in the ranking. On the basis of PageRank, LeaderRank [22] introduces a ground node *g*, which has two directed links to every node in the original network, so that the network becomes strongly connected. LeaderRank converges faster, since the network is strongly connected. The results showed that LeaderRank outperformed PageRank in terms of ranking effectiveness, as well as robustness against manipulations and noisy data. Li et al. [23] improve LeaderRank by introducing a weighted mechanism: nodes with different in-degrees get different ranks from the ground node. In [24], authors define a measure based on topic similarity and structure in the links between users. Influence is considered as the fact of *following* other users regarding topic interests. In this context, the authors propose TwitterRank, an extension of the PageRank algorithm, to measure the topic-sensitive influence of users. Although the idea is promising, the experimental results show that there are some *follow* links between users not because of the topic similarity between them, also the method ignored other important relations, such as *mentions* and *replies*. Ashwini et al. [25] consider that *Twitter* is a platform of information diffusion and study the problem of identification of influential users. They propose ProfileRank, an information diffusion mode based on random walks that estimates users influence. ProfileRank is based on the principle that an influential user creates a pertinent content. The limit of this approach is that influence is assessed based only on the *retweet* relation and the method ignores the other relations.

In the same context of the diffusion-based algorithms, some researchers proposed variants of the HITS algorithm (hyperlink-induced topic search), a link analysis algorithm that rates Web pages, developed by Jon Kleinberg. HITS assigns two scores for each page: its authority, which estimates the value of the content of the page, and its hub value, which estimates the value of its links to other pages. Romero et al. [26] propose the IP-algorithm to measure influence. In this paper, influence is considered as the degree of content propagation in the network (*retweets*). In addition, authors believe that a user’s influence depends not only on the size of the influenced audience, but also on their passivity. The passivity of a user is his passive information consuming without forwarding the content to the network. The algorithm showed better accuracy than other influence measures, such as PageRank, the number of *followers* and number of *mentions*. Although passivity seems a good influence indicator, this work ignored other important relation such as *reply*. The diffusion-based algorithms, such as variants of the PageRank and HITS, were designed considering the information propagation in the network. Their shortcoming is the lack of relations and interactions combination.

In recent works, **information fusion** is considered to address limitations of existing methods. In [27], authors propose a combination of two models for ranking users’ influence: The PageRank algorithm [11] and HMM (hidden markov model). They build a HMM to observe the influence evolution over time and use three relations: *retweet*, *mention*, and *reply*. The model is evaluated using a survey as ground-truth for influence ranking. The proposed model differs from the others by combining the important relations. However, as the purpose is to rank users’ influence, a user’s given influence does not reveal information about its influence degree (high or low influence), and the model’s output is only useful in users ranking. Moreover, the authors do not offer a measure of influence by exploiting the combination of criteria with its inherent uncertainty. However, it seems important to consider the degree of uncertainty on the weights assigned to the different relations and interactions according to their importance.

In this purpose, recent research uses the belief functions theory to assess user’s influence in weighted networks [28, 29] and complex networks [30]. To the best of our knowledge, this is the first time belief functions theory is exploited to assess influence on *Twitter* network using different forms of interactions instead of centrality measures.

### Belief functions theory

Every day, a huge volume of incomplete and imperfect information is produced by social networks applications. Thus, reasoning with uncertainty has become a major interest in the analysis of social networks data.

The belief functions theory is considered as a general framework for reasoning with uncertainty, and has well been connected to other frameworks, such as probability, possibility, and imprecise probability theories [31]. The theory of belief functions, also known as evidence theory or Dempster–Shafer theory, was first introduced by Dempster in the context of statistical inference, and was later developed by Shafer as a general framework for modeling epistemic uncertainty, which means, due to a lack of knowledge [32].

In the following, we are going to remind the basic concepts of belief functions theory. Let $\Omega$ be a finite set, denote by $2^\Omega$ the set of all subsets of $\Omega$. In the context of Dempster–Shafer theory, $\Omega$, often called a frame of discernment, represents the set of possible answers to a certain question. A mass *m* is a function $m\,{:} \,2^\Omega \longrightarrow [0,1]$ such that:

1 $$\begin{aligned} \underset{X\in 2^\Omega }{\sum }m(X) = 1 \; \text {and} \; m(\emptyset )=0 \end{aligned}$$

The mass *m*(*X*) expresses the part of belief that supports the subset *X* of $\Omega$ and $m(\Omega )$ represents the degree of ignorance. According the theory of closed-world, $\Omega$ is exhaustive, and hypotheses are mutually exclusive and $m(\emptyset )=0$.

Belief functions theory allows not only the representation of the partial knowledge, but also the information fusion under uncertainty [33]. Considering different sources of information expressed on the same frame of discernment, we would like to combine these information through one single belief mass. This is done by the conjunctive combination rule [7], and it assumes that all sources are reliable and consistent. Considering two mass functions $m_1$ and $m_2$, the conjunctive combination rule is defined as:2To make a decision, we try to select the most likely hypothesis which may be difficult to realize directly with the basics of the belief functions theory where mass functions are given not only to singletons but also to subsets of hypothesis. There exist several solutions to ensure decision making within the belief functions theory. The most known is the pignistic probability [34]. In contrast to mass functions that are defined on $2^\Omega$, pignistic probability is a probability measure defined on $\Omega$. Pignistic probability was proposed in the transferable belief model (TBM) [35]. It is based on two levels: The “credal level” where beliefs are entertained and represented by belief functions and the “pignistic level” where beliefs are used to make decisions and represented as probability functions called pignistic probabilities denoted bet:

3 $$\begin{aligned} \mathrm {bet}(x)=\sum _{x \in X \subseteq 2^\Omega } \frac{m(X)}{|X|} \end{aligned}$$

## Proposed approach

**Fig. 1:**
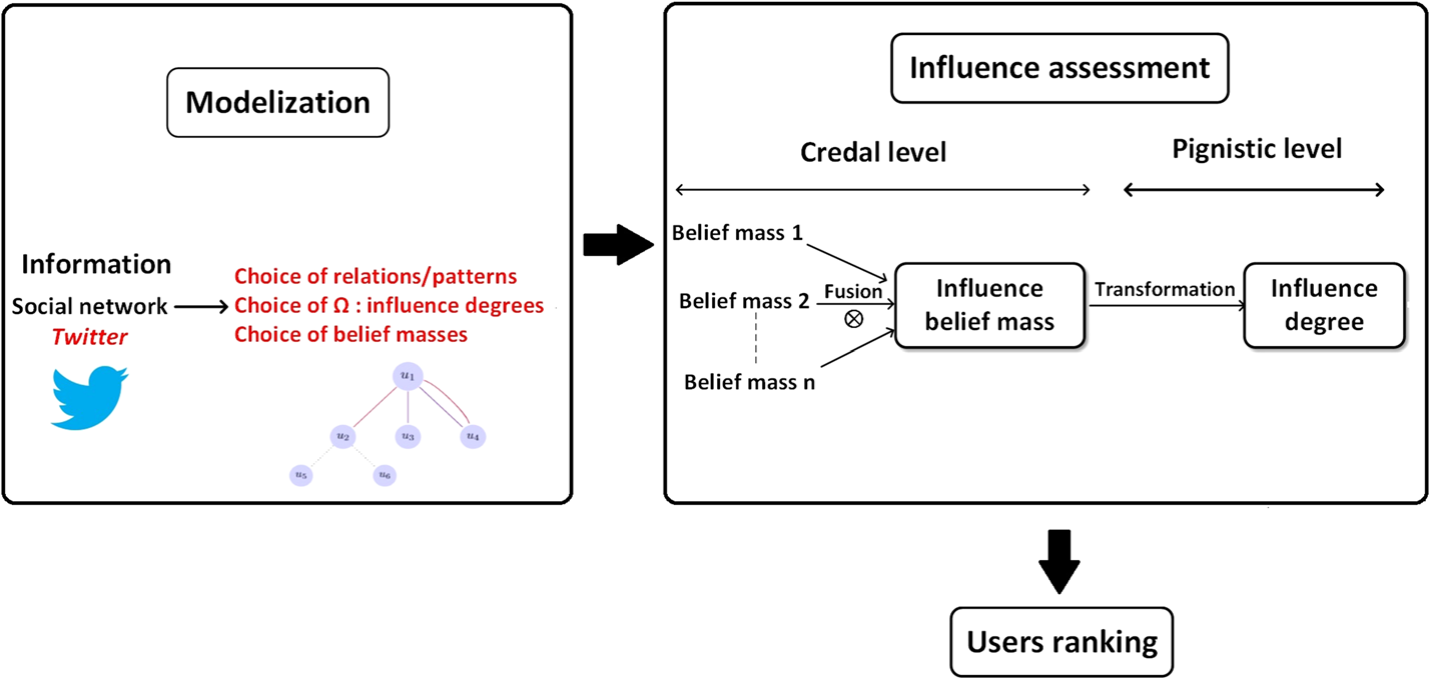
Framework of the proposed approach

To assess users’ influence, we propose a belief approach based on information fusion about the different possible influence relations or interaction forms (simple or complex interaction patterns). Figure 1 shows an overview of the framework of the proposed approach. In the first step, information from *Twitter* network is gathered and modeled in a graph by selecting relevant relations or patterns for the influence model. After that, the choice of influence degrees and belief masses initialization are performed. The next step is the influence assessment: first, at a credal level, we combine belief masses associated to each considered relation or interaction pattern to obtain the influence belief mass. At the pignistic level, we compute the pignistic probability to make a decision about the user’s influence degree. Finally, based on the influence degree of each user, we rank all users. In the following sections, we detail each step of the assessment process.

### Modelization

Social networks have been widely modeled as a graph [36]. A graph is represented as $G=(V,E)$ comprising a set *V* of vertices or nodes together with a set *E* of edges or links. In *Twitter* network, the graph is heterogeneous as we have many relations between nodes and different types of nodes. For example, there may exist a link *follow* between two users, a link *retweet* between one *tweet* and a user. To model this heterogeneity, we use a multi-relational graph [4, 37, 38]. As we want to evaluate a user’s influence on other users, we restrict the graph to homogeneous nodes (users), and thus, we have a multiplex graph (sometimes called multi-layered graph) [39]. In multiplex graphs domain, new measures and methods have been proposed in the literature to analyze these networks. The most known is the edge entanglement in multiplex networks [40] which allows a better understanding of multiplex networks.

In a multiplex graph, the set of edges *E* is divided into pairwise disjoint classes $E = \bigcup _{r\in R} E_r$, where *R* is the set of possible relations. We define an interaction pattern *p* as a sequence of relations, for example, a *retweet* of a *reply* or *retweet* of a *tweet* with a *mention*. Let *P* the set of all the interaction patterns that have been identified for modeling influence in a specific domain or for a specific study. This set can be given by social scientists for example. We denote by $R = R \bigcup P$ the set of relations including interaction patterns. For example, in *Twitter* we can consider:

R = {*Follow*,  *Retweet*,  *Mention*,  *Reply*,  *Like*,  *Retweet of Reply*,  *Retweet of Mention*}

Our goal is to assess users influence. So, $\Omega$ represents the different possible answers to our question: What is the influence degree of a certain user? Let $\Omega$ be an ordered set of possible influence degrees:

4 $$\begin{aligned} \Omega &= \{\textsf {Very Weak},\, \textsf {Weak},\, \textsf {Average Enough},\, \textsf {Average},\, \textsf {Strong Enough},\, \nonumber \\ &\textsf {Strong},\, \textsf {Very Strong},\, \textsf {Extremely Strong}\} \end{aligned}$$

In our approach, relations are the manifestation criteria of a user’s influence. Hence, a user’s influence is determined by the importance of his related relations. Each relation is associated with an influence degree $d_r$ for $r\in R$, for example, the relation *retweet* is associated with the influence degree $d_{retweet} = V.Weak$. In general Dempster–Shafer theory, $2^\Omega$ is used as a domain of mass functions. But in our approach, we only use a certain subset $\Lambda$ of $2^\Omega$, because we want to translate the expert’s certainty by a mass function *m* on a relation, precisely:

5 $$\begin{aligned} \Lambda &= \{\textsf {Very Weak},\, \textsf {Weak},\, \textsf {Average Enough},\, \textsf {Average},\, \textsf {Strong Enough},\, \nonumber \\ &\textsf {Strong}, \textsf {Very Strong}, \textsf {Extremely Strong}, \Omega \} \end{aligned}$$

A mass function is associated for each relation, and therefore, mass functions are defined as follows: $m_r\,{:}\,\Lambda \rightarrow [0,1]$.

**Fig. 2:**
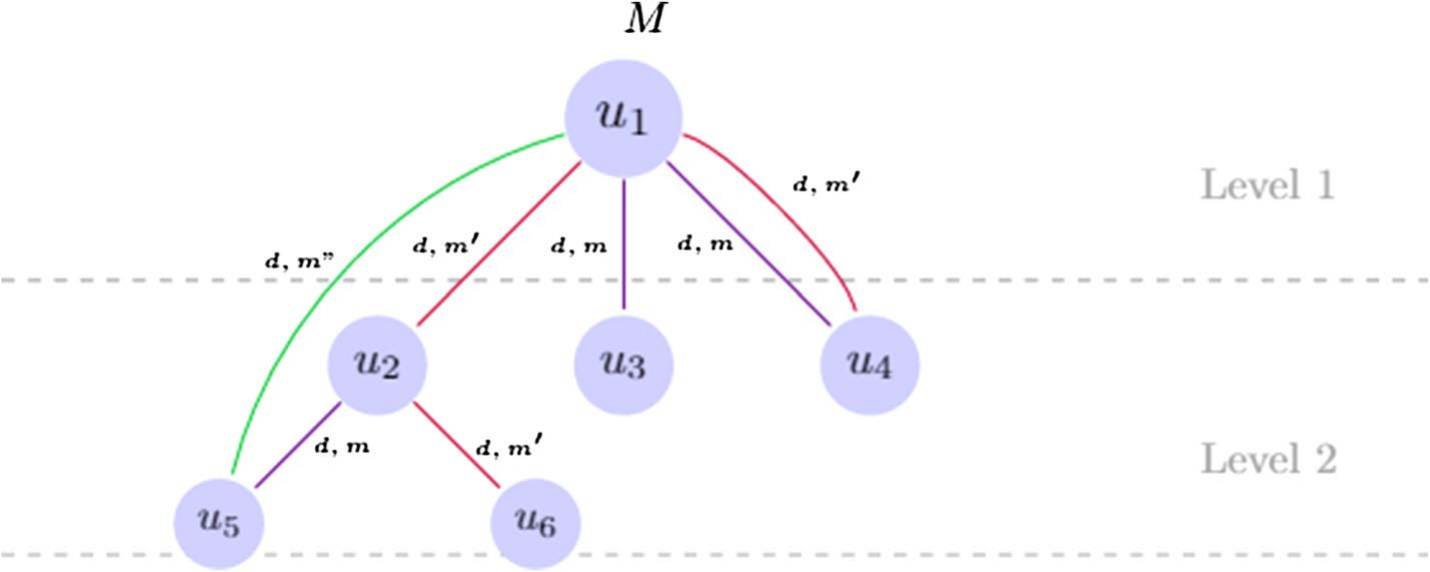
Influence graph

Hence, for each relation type $r \in R$, in addition to the influence degree $d_r$, a mass function $m_r$ is associated. In this context, we introduce the *influence graph* (Fig. 2) as a labeled multiplex graph $G=(U,E)$, where *U* is the set of nodes represented by users, and *E* is the set of links that model the different relations $r \in R$ between nodes. The links are labeled with influence degrees (e.g., Weak, Average, and Strong) and belief masses $m_r$ that depend on the type of the relation. Nodes are labeled with their estimated influence degree resulting from the fusion of the belief masses of incident links (denoted by *M* in the Fig. 2). Some recent researches have introduced uncertain graphs whose edges are labeled with a probability of existence [41, 42]. In our case, uncertainty is not about the presence or absence of links but is about the our belief in the importance weight of links according to the domain. For example, in political studies, a *mention* or a reply can be less valuable than a *retweet*, and also a *reply* followed by a *retweet* is a very important interaction pattern.

### Influence assessment

#### Masses fusion on the influence graph

Based on the belief functions theory discussed above in the "Literature review" section, we explain how to make the fusion of different mass functions defined on the influence graph. To estimate the influence degree of a specific node $u \in U$, we take into account the local structure of the influence graph around the node *u* and combine the belief mass functions of incident links using a modified version of conjunctive combination rule (2).6
 is a symmetric function, . Table 1 shows an example of an  function. This function assures our hypothesis: the more we combine relations about a user, the more his influence becomes important.

**Table 1:** Definition of the function

	V.Weak	Weak	Average.E	Average	Strong.E	Strong	V.Strong	E.Strong	${\Omega }$
V.Weak	Weak	Average.E	Average	Strong.E	Strong	V.Strong	V.Strong	E.Strong	V.Weak
Weak	Average.E	Average.E	Average	Strong.E	Strong	V.Strong	V.Strong	E.Strong	Weak
Average.E	Average	Average	Strong.E	Strong	V.Strong	V.Strong	V.Strong	E.Strong	Average.E
Average	Strong.E	Strong.E	Strong	Strong	V.Strong	V.Strong	V.Strong	E.Strong	Average
Strong.E	Strong	Strong	V.Strong	V.Strong	V.Strong	V.Strong	V.Strong	E.Strong	Strong.E
Strong	V.Strong	V.Strong	V.Strong	V.Strong	V.Strong	E.Strong	E.Strong	E.Strong	Strong
V.Strong	V.Strong	V.Strong	V.Strong	V.Strong	V.Strong	E.Strong	E.Strong	E.Strong	V.Strong
E.Strong	E.Strong	E.Strong	E.Strong	E.Strong	E.Strong	E.Strong	E.Strong	E.Strong	E.Strong
$\Omega$	V.Weak	Weak	Average.E	Average	Strong.E	Strong	V.Strong	E.Strong	$\Omega$

Next, we discuss two important properties of the generalized combination rule $\otimes$ and the symmetric function  that replaces the intersection operator in the classical rule.

##### **Proposition 1**

*A combination of any two mass functions is another mass function.*

##### *Proof*

Denote $(m \otimes m')$ by $m''$. It is easy to see that for all *x* we have $m''(x)\, \geqslant \, 0$, because we compute $m''$ using only multiplication and addition of non-negative numbers. Next, we show that  and proceed as follows:Note that $\Lambda _z^2\ne \Lambda _{z'}^2 \iff z \ne z'$, and $\bigcup _{z \in \Lambda } \Lambda _z^2 = \Lambda ^2$. So, we can ommit $\sum _z$ and rewrite as follows:

$$\begin{aligned} &= \sum \limits_{(x,y) \in \Lambda ^2} m(x) m'(y) \\ &= \sum \limits_x \sum \limits_y m(x) m'(y) \\ &= \sum \limits_x m(x) \sum \limits_y m'(y) \end{aligned}$$

*m* and $m'$ are mass function: $\sum _x m(x) = \sum _y m'(y) = 1$, so $\sum _x m(x) \sum _y m'(y) = 1.$ $\square$

##### **Proposition 2**

*In general* $\otimes$ *is non-associative:* $(m \otimes m') \otimes m'' \ne m \otimes (m' \otimes m'')$

##### *Proof*

Consider $\Omega =\{A,B,C\}$, and the following  : 
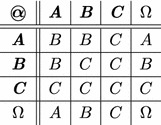

$$\begin{aligned} m = m'&= \begin{array}{c|c|c|c|c} &{} A &{} B &{} C &{} \Omega \\ \hline &{} 1 &{} 0 &{} 0 &{} 0 \end{array} \\ m ''&= \begin{array}{c|c|c|c|c} &{} A &{} B &{} C &{} \Omega \\ \hline &{} 0 &{} 1 &{} 0 &{} 0 \end{array} \end{aligned}$$

It is easy to see that:

$$\begin{aligned} (m \otimes m') \otimes m''&= \begin{array}{c|c|c|c|c} &{} A &{} B &{} C &{} \Omega \\ \hline &{} 0 &{} 0 &{} 1 &{} 0 \end{array} \\ m \otimes (m' \otimes m'')&= \begin{array}{c|c|c|c|c} &{} A &{} B &{} C &{} \Omega \\ \hline &{} 0 &{} 1 &{} 0 &{} 0 \end{array} \end{aligned}$$

Thus, in general:

$$\begin{aligned} (m \otimes m') \otimes m'' \ne m \otimes (m' \otimes m'') \end{aligned}.$$

$$\square$$

As multiple relations can exist between a node *u* and its neighbors, we combine the associated mass functions $m_r$ for each relation $r \in R$. We denote by $I_r$ the set of all links with relation type *r*. Finally, we have the following set of mass functions $\{ m_{r,i} : r \in R, i \in I_r\}$. Based on Proposition 1, we can combine these mass functions to obtain a global belief mass corresponding to the influence degree of node *u*. However, the order of combinations may affect our results (Proposition 2). To be consistent in our measurements, we have to fix the order of combinations and masses involved in these combinations. To simplify the expressions, we write $\bigotimes _{i \in \{1,2,3,\ldots \}}$ instead of $\Big ( \big ( (m_1 \otimes m_2 )\otimes m_3 \big ) \ldots \Big )$. Thus, we consider the following order of combinations:

1. For a given relation *r*, we subsequently combine the masses of *r* to get *r*-preresult with $\hat{m}_r$ defined as follows: $\displaystyle \hat{m}_r = \bigotimes _{i \in I_r} m_{r,i}$.
2. Then, we combine all *r*-preresults using: $\displaystyle \bigotimes _{r \in R} \hat{m}_r$.

Depending on the function  such procedure may finally converge to certain stationary mass.

#### Pignistic probability transformation

Once we have the global belief mass on a certain node, we use a modified version of the pignistic probability defined by Eq. (3) to make the decision about the influence degree of a user. In our case, the belief masses are defined on $\Lambda$ and the pignistic probability is calculated by distributing uniformly the ignorance mass $m(\Omega )$ to all other elements of $\Lambda$:

7 $$\begin{aligned} \mathrm {bet}(x)= m(x) + \frac{m(\Omega )}{|\Omega |},\quad x \in \Lambda \setminus \{\Omega \} \end{aligned}$$

The process of the influence evaluation step is described in Algorithm 1, and the algorithm requires as input, the multiplex influence graph $G = (U,E)$ defined as above, the masses and influence degree initialization for the different relations $m_r, r \in R$, and the function  . For each user, the algorithm starts by counting the number of occurrences for each relation or interaction pattern. Then, for each relation *r* type, using Eq. (6), it computes the belief masses combination. After that, Eq. (6) is used again to combine the belief masses for all relations. And finally, using Eq. (7), the belief masses distributions are transformed to pignistic probability. The algorithm returns the final influence degree which is the degree having the maximal pignistic probability. The set of final influence degrees $\{ {{\mathrm{Inf}}}_u : u \in U\}$ is denoted by ${{\mathrm{Inf}}}$. The source code is available on github.^1^ It is the general R code that can be specialized depending on the studied network and used relations.

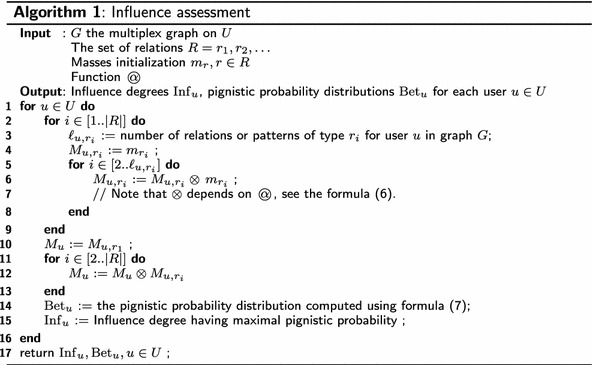



To discuss the complexity of Algorithm 1, we have to determine the complexity of the combination rule (6) and the pignistic probability (7). The complexity of a fusion operator (6) is $O(|\Lambda |^2)$ in general, because it corresponds to the matrix-vector multiplication [43]. Let $d_u$ be the number of relations of any type that involve a user *u*. Let $\Delta = \max {u \in U} d_u$. Thus, the maximum number of combinations to compute ${{\mathrm{Inf}}}_u$ is $\Delta -1$ for any user *u*. Next, we have |*U*| users in total. So, the complexity of Algorithm 1 is $O(|U|\Delta |\lambda |^2 )$, since the pignistic probability distribution is computed in $O(\Lambda )$ operations and the influence degree is also computed in $O(\Lambda )$ operations. In the case of this paper, $|\Lambda |$ is fixed the complexity can be writed as $O(|U| \Delta )$.

#### Illustrations

To illustrate the previous discussed steps of our method, we consider the following mass functions initialization associated to the relations *retweet* and *mention*:

$$\begin{aligned} \text {Retweet} \mapsto \left\{ \begin{array}{ll} m_{\textsf {retweet}} (\textsf {Weak}) &{} =0.4 \\ m_{\textsf {retweet}} (\Omega ) &{} = 0.6 \end{array}\right.&\quad \textsf {Mention} \mapsto \left\{ \begin{array}{ll} m_{\textsf {mention}} (\textsf {V.Weak}) &{} =0.3 \\ m_{\textsf {mention}} (\Omega ) &{} = 0.7 \end{array}\right. \end{aligned}$$

The belief masses $m_{\textsf {retweet}} (\Omega )$ and $m_{\textsf {mention}} (\Omega )$ represent the partial ignorance.

##### ** Case 1: Two retweets**

After initialization of belief masses on the different relations, we follow the proposed approach process to measure the influence resulted from combination of two *retweets* from one user to another. We first use the function  giving the correspondences between the influence degrees, then we calculate the conjunctive combination. The combined mass function of the two *retweets* are shown in Table 2:

**Table 2:** Combination of two *retweets*

${\otimes }$	Weak	$\Omega$
	0.4	0.6
Weak	Average.E	Weak
0.4	0.16	0.24
$\Omega$	Weak	$\Omega$
0.6	0.24	0.36

We obtain then:

$$\begin{aligned} m(\textsf {Weak})= & \; 0.24 + 0.24 \, = \; 0.48\\ m(\textsf {Average.E})= & \; 0.16 \\ m(\Omega )= & \; 0.36 \end{aligned}$$

Finally, to make a decision about the influence degree, we calculate the pignistic probability using Eq. (7) (see Table 3). For example, for the degree Weak, we proceed as follows to obtain the pignistic probability:

$$\begin{aligned} \mathrm {bet}(\textsf {Weak})= m(\textsf {Weak}) + \frac{m(\Omega )}{|\Omega |} = 0.48 + \frac{0.36}{8} = 0.525 \end{aligned}$$

**Table 3:** Pignistic probability for two *retweets*

V.Weak	0.045
Weak	0.525
Average.E	0.205
Average	0.045
Strong.E	0.045
Strong	0.045
V.Strong	0.045
E.Strong	0.045

We conclude that the influence degree is Weak since it has the highest pignistic probability 0.525. This latter was 0.4 before considering the combination.

##### **Case 2: 2 retweets + 2 mentions**

In the second case, we consider two additional *mentions* existing between the same users of case 1. To measure influence, we use our proposed process to combine masses of the two *mentions*, and then, we combine the obtained masses with the results of the previous case related to the two *retweets* combination (Tables 4, 5). The conjunctive combination on the two *mentions* gives:

**Table 4:** Combination of two *mentions*

${\otimes }$	V.Weak	$\Omega$
	0.3	0.7
V.Weak	Weak	V.Weak
0.3	0.09	0.21
${\Omega }$	V.Weak	$\Omega$
0.7	0.21	0.49

We obtain:

$$\begin{aligned} m(\textsf {V.Weak})= & \; 0.42\\ m(\textsf {Weak})= & \; 0.09\\ m(\Omega )= & \; 0.49 \end{aligned}$$

Now, we combine the obtained masses with the results of case 1:

**Table 5:** Case 2: 2 *retweets* + 2 *mentions*

${\otimes }$	Weak	Average.E	${\Omega }$
	0.48	0.16	0.36
V.Weak	Average.E	Average	V.Weak
0.42	0.2016	0.0672	0.1512
Weak	Average.E	Average	Weak
0.09	0.0432	0.0144	0.0324
${\Omega }$	Weak	Average.E	$\Omega _{Inf}$
0.49	0.2352	0.0784	0.1764

We obtain:

$$\begin{aligned} m(\textsf {V.Weak})= & \; 0.1512\\ m(\textsf {Weak})= & \; 0.2676\\ m(\textsf {Average.E})= & \; 0.3232\\ m(\textsf {Average})= & \; 0.0816\\ m(\Omega )= & \; 0.1764 \end{aligned}$$

We note that, by combining the four relations, the belief mass on the degree Weak has decreased compared to the first case, and this is due to the fact that the mass of the Average.E degree has increased and became equal to 0.3232. We also notice that the degree Average appeared with a mass equal to 0.0816. We can conclude that the more we have relations and the more we combine them, the highest influence we get. Now, to make the decision about the influence degree, we compute the pignistic probability (Table 6). We conclude that the influence degree for two *retweets* and two *mentions* is Average.E with a pignistic probability of 0.34525. This latter was 0.205 before considering the two *mentions*.

**Table 6:** Pignistic probability for case 2

V.Weak	0.17325
Weak	0.28965
Average.E	0.34525
Average	0.10365
Strong.E	0.02205
Strong	0.02205
V.Strong	0.02205
E.Strong	0.02205

### Users ranking

In this step, we exploit results of the influence assessment to rank users according to their influence. Algorithm 2 describes the used method to rank users’ influence. First, for each user, we take the influence with maximal pignistic probability (for example, Inf(“$U_1$” = E.Strong). After that, we rank users by their “maximal influence degree.” When two users have the same “maximal influence degree”: $Inf({``U_1''}) = \textsf {V.Strong}$ and $Inf({``U_2''}) = \textsf {V.Strong}$ we compare belief masses of the next-greater influence degree and rank them according to the next-greater influence degree.

We use the following order of influence degrees ranking:

$$\Omega< \textsf {V.Weak}< \textsf {Weak}< \textsf {Average.E}< \textsf {Average}< \textsf {Strong.E}< \textsf {Strong}< \textsf {V.Strong} < \textsf {E.Strong}$$

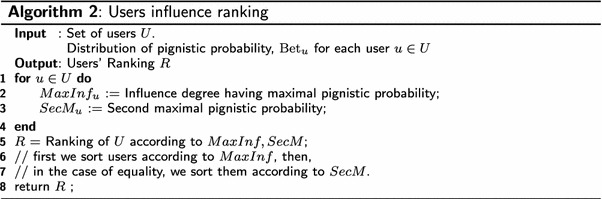



For computing $MaxInf_u$ and $SecM_u$ for one user *u*, we need to perform $O(|\Lambda |)$ operations. We have |*U*| users, so, lines 1–3 take $O(|U||\Lambda |)$ operations. After that, we sort the set of users. Thus, the total complexity of Algorithm 2 is $O\big (|U| ( |\Lambda | + log |U| ) \big )$.

We proceed this way, since it is unfair to rank users by maximal belief masses they have on the degrees. This is because during the process of the masses fusion, a user’s influence increases and passes from an influence degree to the next greater degree and so on. Thus, for users who have many combinations, they pass from the weaker influence degree (V.Weak) until they reach high influence degrees. Therefore, the masses on each degree starts weak, and as we combine, this mass becomes more important, and then it decreases and the mass on the next greater influence degree increases instead. For this reason, to rank users who have maximal belief mass on the same degree (for example, two users who have the degree V.Strong as a maximal degree), we should not consider the mass they have on this degree, because we may have a user more influential than another, although he has a weaker belief mass than him on the same degree. This is due to the fact that, the belief mass on the next-greater degree has increased and became quite important. Hence, to compare users who have maximal belief mass on the same degree, we should rather consider the belief mass on the next greater degree.

## Experiments and results

To evaluate our approach, we use two data sets, the TEE 2014 data set and the CLEF Replab 2014 data set.

### TEE 2014 Data set

Our research work takes place in the project TEE 2014 whose exact title is: “*Twitter* in the European Elections: an international contrasting study of *Tweets* use by candidates in elections to the European Parliament in May 2014.” This international project led by the House of Human Sciences (MSH) in Dijon, brings together nearly 45 researchers (political scientists, sociologists, communication researchers and computer scientists), 10 research laboratories spread across 6 European countries (France, Germany, Belgium, Italy, Spain and the UK). The overall objective of this project is to observe and analyze the *Tweets* communication policies during the election period in May 2014 in various countries of Western Europe.

#### Data description

The *tweets* collection during the election period has build a corpus which is then analyzed. To collect information from *Twitter*, we used our developed tool *SNFreezer* 2 [44]. The purpose of gathering is to retrieve *tweets mentioning* designated users, those containing a *hashtag*, a word or phrase related to the European Elections (e.g., the hashtag #UE14), or *tweets* sent by candidates. Three types of information (generalized under the term “source”) are taken as a parameter to query *Twitter*: user accounts; *hashtags* and words or phrases.

These sources were chosen by political scientists, and among them we find the names of the leading candidates, their *Twitter* accounts and their parties. The collection allowed us to have a large number of *tweets* (37 million) retrieved for 50 consecutive days, and to massively process these data. In our experiments, we focus on the French corpus. Table 7 shows the parameters of the used data set.

**Table 7:** Parameters of the French corpus TEE 2014 data set

Number of tweets	4,593,665
Number of nodes	937,860
Number of candidates	616
Number of edges	2,922,566
Number of retweets	639,531
Number of mentions	1,945,773
Number of replies	337,262

#### Modelization

Our experimental goal is to measure and rank candidates influence on the network. Unlike illustrations given in the previous section, we do not consider the case of measuring influence between two users but rather global candidates’ influence in the network. Also, the measure takes into account both the direct and indirect influence. The direct influence (first-level influence) takes into account only the direct links between the users in the influence graph. Relations representing the direct influence are *retweets*, *mentions*, and *replies*. In the indirect influence, we can consider the *retweets* of *replies* and the *retweets* of *mentions*. Those relations are called indirect ones as they are performed on indirect nodes in the network (e.g., a user may *retweet* another’s *tweet* indirectly through an intermediate user). The indirect influence is a more complex form of dialog compared to the direct influence. Relations or patterns are determined depending on the domain.

**Fig. 3:**
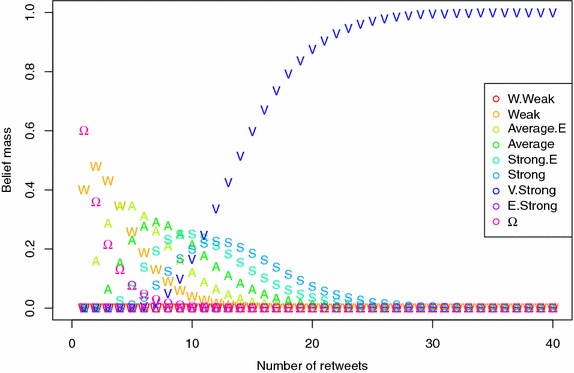
Influence rapidly converges to the highest possible degree when the number of *retweets* grows

The choice and the affectation of the masses in the initialization step is an important issue while dealing with real data. In some domains, such as politics, users have very high number of relations. With masses initialized as in the illustration’s section, influence rapidly converges to the highest possible degree E.Strong (after only 40 *retweets* combination). Figure 3 shows this rapid convergence when the belief mass of a *retweet* is defined as $m_{\textsf {retweet}} (\textsf {Weak})=0.4$, $m_{\textsf {retweet}} (\Omega ) = 0.6$. In [43], authors study in details several theoretical questions about the convergence using the Markov chain theory.

In this way, we cannot compare candidates’ influence as we obtain the same influence degree with similar masses for most of them. To deal with this, we perform a rescaling and use the following masses initialization:

$$\begin{aligned} Retweet \mapsto \left\{ \begin{array}{l} m_{\text {retweet}} (\text {V.Weak}) = 0.55 \cdot 10^{-3} \\ m_{\text {retweet}} (\Omega ) = 1 - 0.55 \cdot 10^{-3} \end{array}\right. \end{aligned}$$

$$\begin{aligned} Mention \mapsto \left\{ \begin{array}{l} m_{\text {mention}} (\text {V.Weak})= 0.45 \cdot 10^{-3}\\ m_{\text {mention}} (\Omega ) = 1 - 0.45 \cdot 10^{-3} \end{array}\right. \end{aligned}$$

$$\begin{aligned} Reply \mapsto \left\{ \begin{array}{l} m_{\text {reply}} (\text {V.Weak})= 0.45 \cdot 10^{-3}\\ m_{\text {reply}} (\Omega ) = 1 - 0.45 \cdot 10^{-3} \end{array}\right. \end{aligned}$$

$$\begin{aligned} Retweet \, of \, Reply \mapsto \left\{ \begin{array}{l} m_{\text {retweetOFreply}} (\text {V.Weak})=0.75 \cdot 10^{-3} \\ m_{\text {retweetOFreply}} (\Omega ) = 1 - 0.75 \cdot 10^{-3} \end{array}\right. \end{aligned}$$

$$\begin{aligned} Retweet \, of \, Mention \mapsto \left\{ \begin{array}{l} m_{\text {retweetOFmention}} (\text {V.Weak})= 0.65 \cdot 10^{-3}\\ m_{\text {retweetOFmention}} (\Omega ) = 1 - 0.65 \cdot 10^{-3} \end{array}\right. \end{aligned}$$

The mass of the *retweet* relation is more important than the others, because we consider that it is a better influence indicator than the other relations. Also, the masses of the indirect relations are a little more important than those initiated for the direct influence relations, because we consider that indirect interactions are good indicator of influence, and this shows that some users are able to diffuse *tweets* on many levels and they are exercising influence even on users with which they are not connected.

#### Influence assessment

To measure influence on the first level, we take each candidate’s number of *retweets*, *mentions*, and *replies* and combine their masses. Table 8 show the first-level combination results for the candidates “Marine Le Pen,” “Florian Philippot,” and “Jean-Luc Mélenchon.” For example, we conclude that the influence degree for the candidate “Marine Le Pen” who has 14,678 *retweets*, 66,798 *mentions*, and 4003 *replies* is E.Strong with the belief mass of 0.8173448. Results given do not only provide the influence degree but also give indication of our belief in the given results which is performed by the belief masses on the different degrees.

**Table 8:** Results for the top 3 influential French candidates

	M. Le Pen	F. Philippot	J. L. Mélenchon
E.Weak	0	0.000011065	0.000030278
Weak	0	0.00007295998	0.0001832843
Average E	0	0.0007035528	0.001403947
Average	0	0.003033557	0.004954501
Strong.E	0	0.008340205	0.01247841
Strong	0	0.02191526	0.02977818
V.Strong	0.1826552	0.5830090	0.7960571
E.Strong	0.8173448	0.3829144	0.1551143

In the previous results, we only considered direct influence for the candidates. The proposed approach is also flexible and can be extended to multi-level belief fusion using interaction patterns. To consider indirect influence in our assessment, we evaluate the indirect influence and then combine the results with those obtained from the direct influence.

Table 9 represents the results of the multi-level belief fusion for top 3 influential French candidates. It shows that influence has became more important after considering the indirect influence. For example, for the candidate “Marine Le Pen,” we found that she has 4003 *retweets of replies* and 37,715 *retweets of mentions*, the influence degree obtained after multi-level fusion is still the same degree E.Strong, but the belief mass has became more important and has reached 0.99.

**Table 9:** Results for the top 3 influential French candidates according to multi-level fusion

	Marine Le Pen	Christine Boutin	J.L. Mélenchon
E.Weak	0	0	0
Weak	0	0	0
Average E	0	0	0.00001235114
Average	0	0	0.00009165825
Strong.E	0	0	0.0003897511
Strong	0	0	0.001821524
V.Strong	0.004328	0.0667283	0.345777616
E.Strong	0.995672	0.9332717	0.6519071

#### Users ranking

In this section, our experimental goal is to detect most influential candidates on the network based on our proposed approach. As described in the previous section, we take first, for each candidate, the influence with maximal pignistic probability (for example, Inf(“Marine Le Pen” = E.Strong). After that, we rank candidates by their “maximal influence degree.” When two candidates have the same “maximal influence degree”:

$$\begin{aligned} Inf(\text {``Florian Philippot''}) = \textsf {V.Strong} \end{aligned}$$

$$\begin{aligned} Inf(\text {``Jean-Luc Melenchon''}) = \textsf {V.Strong} \end{aligned}$$

we compare belief masses of the next-greater influence degree:

$$\begin{aligned} m_\text {Philippot}(\textsf {E.Strong}) > m_\text {Melenchon}(\textsf {E.Strong}) \end{aligned}$$

As mentioned before, we proceed this way since it is unfair to rank users by maximal belief masses they have on the degrees. For example, the candidate “Florian Philippot” has a belief mass on the degree V.Strong weaker than the belief mass of “Jean-Luc Mélenchon” on the same degree as we can see in Table 8. Inspite of this, he is ranked before Jean-Luc Mélenchon (Table 10) as he has a greater belief mass on the degree E.Strong. We deduce all the candidates ranking by influence degree. Findings are shown in Table 10. The results are general, taking into account the possible relations and patterns in one same measure.

**Table 10:** Top influential candidates according to belief fusion

Rank	Candidates	Influence degree	Belief mass
1	Marine Le Pen	E.Strong	0.8173448
2	Florian Philippot	V.Strong	0.5830090
3	Jean-Luc Mélenchon	V.Strong	0.7960571
4	Christine Boutin	V.Strong	0.9796956
5	Aymeric Chauprade	V.Strong	0.4171324655
6	Nicolas Dupont-Aignan	V.Strong	0.5293170700
7	José Bové	V.Strong	0.2925722297
8	Geoffroy Didier	Average	0.2092645352
9	Raquel Garrido	Average	0.2048485
10	Marielle De Sarnez	Average.E	0.2074260

**Fig. 4:**
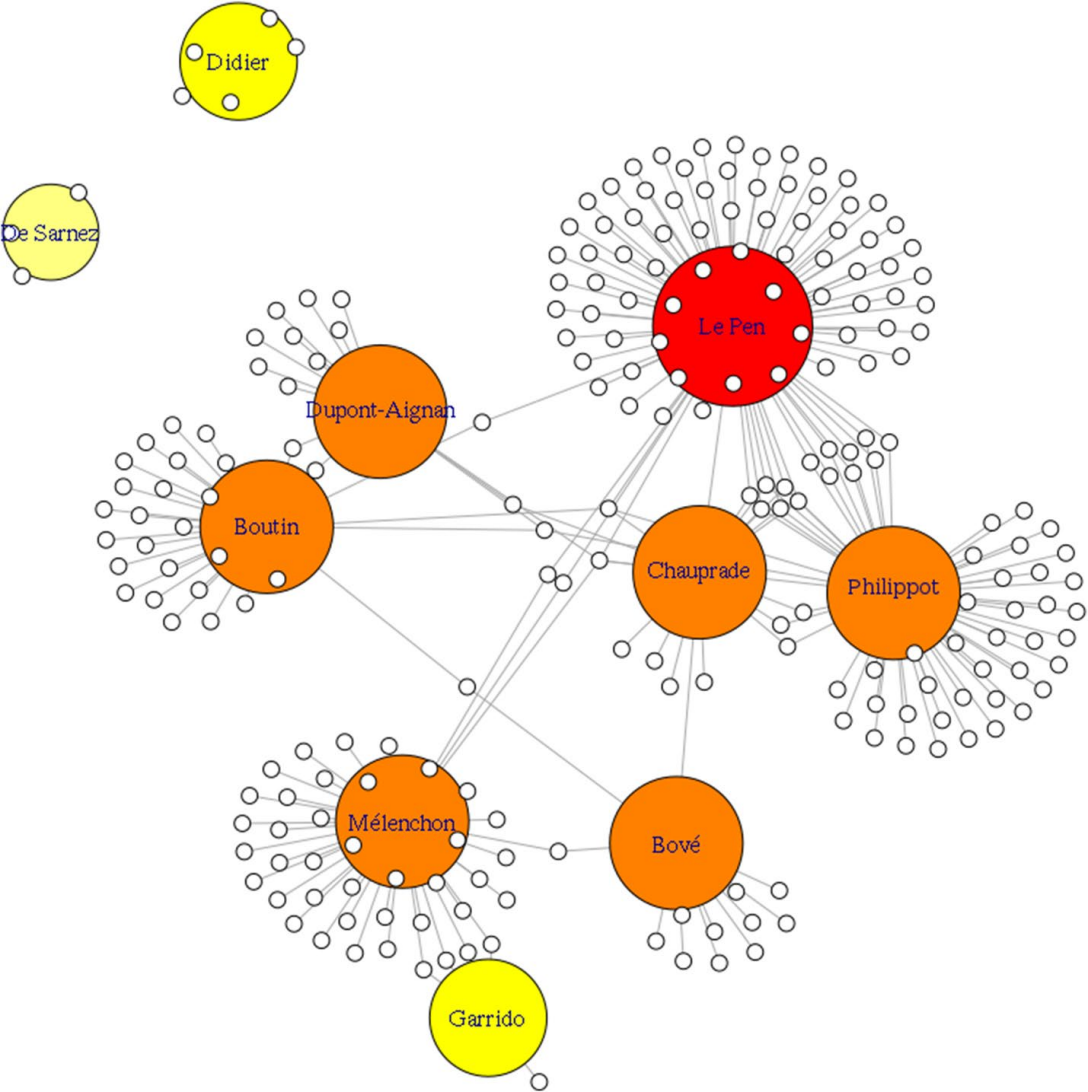
An overview of the influence graph for french candidates

Figure 4 shows a partial visual representation of the diffusion graph corresponding to the French candidates. To bypass the visual complexity of the whole graph, we use only 1 % of all graph data.

Big nodes correspond to candidates accounts, small nodes represent other users. The size and colors of main nodes correspond to their influence degrees: red color corresponds to E.Strong, orange corresponds to V.Strong and yellow is for Average and Average.E degree.

We also ranked the candidates according to multi-level belief fusion. Table 11 shows the new ranking of candidate’s influence after considering indirect influence. Compared to the ranking given in Table 10, some candidate’s ranking has changed. For example, the candidate “Christine Boutin” has became the second most influential candidate as the influence degree increased and became E.Strong with a belief mass of 0.93. This proves the importance of considering the indirect influence in the assessment process.

**Table 11:** Top influential French candidates according to multi-level belief fusion

Rank	Candidates	Influence degree	Belief mass
1	Marine Le Pen	E.Strong	0.995672
2	Christine Boutin	E.Strong	0.9332717
3	Jean-Luc Mélenchon	E.Strong	0.6519071
4	Florian Philippot	E.Strong	0.6021768
5	Aymeric Chauprade	V.Strong	0.66411504
6	Nicolas Dupont-Aignan	V.Strong	0.6615197
7	José Bové	V.Strong	0.6003759
8	Raquel Garrido	V.Strong	0.4805315962
9	Geoffroy Didier	V.Strong	0.416560612
10	Marielle De Sarnez	Strong	0.789653

Table 12 presents the top influential candidates according to the relations considered by Cha et al. in [12] and the interaction patterns. They are ranked by their numbers of *retweets*, *mentions*, *replies*, *retweets of replies*, and *retweets of mentions*. The presented results do not provide the global candidates influence in the network, since different rankings for each relation are given, while our method (Table 10) allows us to to obtain a unique ranking that takes into account all the considered criteria. The first column of Table 13 represent users ranking according to their centrality degree. It is computed using the number of the candidates’ neighbors in the multiplex network. This enables to have a global ranking for the candidates but do not offer any indication on the influence degree of each candidate contrarily to our results that provide influence degrees of each candidate. We also compare our results with those obtained with the HITS algorithm (Table 13), the original algorithm does not combine the different relations and can be based only on one relation, and we have tested the algorithm with each relation separately, namely, *retweets*, *mentions,* and *replies*. The obtained results show different rankings of the candidates’ influence unlike our obtained results.

**Table 12:** Top influential French candidates according to different relations and interaction patterns

Rank	Retweet	Mention	Reply	Retweet of replies	Retweet of mentions
1	M. Le Pen	M. Le Pen	C. Boutin	M. Le Pen	M. Le Pen
2	F. Philippot	C. Boutin	M. Le Pen	C. Boutin	C. Boutin
3	J. Mélenchon	J. Mélenchon	F. Philippot	F. Philippot	J. Mélenchon
4	A. Chauparde	F. Philippot	J. Mélenchon	G. Didier	N. Dupont-Aignan
5	F. Asselineau	N. Dupont-Aignan	L.D.G. Matigon	N. Dupont-Aignan	F. Philippot
6	C. Morel-Darleux	J. Bove	N. Dupont-Aignan	J. Mélenchon	A. Chauparde
7	N. Dupont-Aignan	A. Chauparde	J. Herpin	F. Asselineau	R. Garrido
8	L. Aliot	R. Garrido	J. Rochedy	D.X. Weiss	J. Bove
9	D. Payre	J. Lavrilleux	G. Didier	A. Chauparde	M. de Sarnez
10	Y. Jadot	M. de Sarnez	L. Aliot	J. Rochedy	J. C. Lagarde

**Table 13:** French candidates ranking according to centrality degree and hits algorithm

Rank	Centrality degree	HITS-Reply	HITS-Retweet	HITS-Mention
1	M. Le Pen	M. Le Pen	M. Le Pen	M. Le Pen
2	C. Boutin	F. Philippot	A. Chauprade	A. Chauparde
3	F. Filippot	J.M. Le Pen	B. Monot	F. Philippot
4	J. Mélenchon	G. Didier	F. Philippot	J.M. Le Pen
5	N. Dupont-Aignan	C. Boutin	N. Bay	L. Aliot
6	A. Chauparde	A. Chauparde	B. Gollnisch	B. Monot
7	J. Bove	G. Lebreton	A. Guibert	G. Didier
8	G. Didier	J. Rochedy	G. Lebreton	J. Rochedy
9	R. Garrido	L. Aliot	J. M. Le Pen	G. Lebreton
10	Y. Jadot	N. Bay	K. Ouchikh	B. Gollnisch

#### Discussion

Experiments on the TEE 2014 data set show that the use of our approach leads to interesting results, and the method takes into account different relations and interaction patterns and provides a global influence score in the network. The proposed approach is also flexible and can be extended to multi-level belief fusion using interaction patterns which implies the consideration of indirect influence. The consideration of indirect influence gives different results from the direct influence assessment. These results were appreciated by the sociologists and the political specialists of the TEE 2014 project and indirect influence was judged to be more relevant that direct influence according to the obtained results.

The approach is domain independent and can be applied on any network regardless of the studied domain, and the method requires only the choice of interesting relations and patterns to be considered in the assessment. Also, existing methods simply rank users, our approach is different from these methods as it gives influence score for a given user; after that, the influence score can be used to rank users according to their influence degree.

### RepLab 2014 data set

The CLEF RepLab 2014 data set was designed for an influence challenge organized in the context of the Conference and Labs of the Evaluation Forum (CLEF).^3^ In this subsection, we use this data set for our own experiments.

#### Data description

The RepLab data set contains users manually labeled by specialists from Llorente & Cuenca,^4^ a leading Spanish e-Reputation firm. These users were annotated according to their perceived real-world influence, and not by considering specifically their *Twitter* accounts. The annotation is binary: a user is either influential or not-influential. The data set contains a training set of 2500 users, including 796 influentials, and a testing set of 5900 users, including 1563 influentials. It also contains the 600 last *tweets* ID of each user at the crawling time. These *tweets* could be either written in English or in Spanish. The data set is publicly available.^5^

To evaluate our approach over the RepLab data set, we need to choose relations that will be considered in the assessment, yet the RepLab data set do not provide this kind of information. We only have the users names with 600 *tweets* id for each user. Therefore, we should first gather needed information about these *tweets*; for this, we used Twurl,^6^ a tool that enables to collect information about the given *tweets* from *Twitter* API. However, *Twitter* API limits the collection to 180 *tweets* per 15 minutes. Therefore, it needs 174 days to collect information about all the *tweets*. For this reason, we limit the evaluation on 500 users with 600 *tweets* per user. The relations that we can extract from the collected *tweets* information are: number of *retweets*, *likes,* and *followers*. Table 14 shows the parameters of the used data set.

**Table 14:** Parameters of the RepLab 2014 data set

Number of tweets	300,000
Number of nodes	500
Number of edges	6,769,054
Number of retweets	1,609,438
Number of likes	572,398
Number of follows	4,587,218

#### Experiments

Now, we present our experiments on the RepLab data set. The masses initialization is presented as follows:

$$\begin{aligned} Retweet \mapsto \left\{ \begin{array}{l} m_{\text {retweet}} (\text {V.Weak})=0.55 \cdot 10^{-3} \\ m_{\text {retweet}} (\Omega ) = 1 - 0.55 \cdot 10^{-3} \end{array}\right. \end{aligned}$$

$$\begin{aligned} Like \mapsto \left\{ \begin{array}{l} m_{\text {like}} (\text {V.Weak})= 0.45 \cdot 10^{-3}\\ m_{\text {like}} (\Omega ) = 1 - 0.45 \cdot 10^{-3} \end{array}\right. \end{aligned}$$

$$\begin{aligned} Follow \mapsto \left\{ \begin{array}{l} m_{\text {follow}} (\text {V.Weak})= 0.4 \cdot 10^{-3}\\ m_{\text {follow}} (\Omega ) = 1 - 0.4 \cdot 10^{-3} \end{array}\right. \end{aligned}$$

The most important masses is given to the *retweet* relation, because we believe that this relation is a better influence indicator, the *follow* relation is given the less important belief masses as this indicator gives the popularity of a user, so we use the “popularity” as an influence indicator but we do not give it a very important belief mass. Popular users are not necessarily the most influential ones.

#### Evaluation metric

The RepLab task can be seen as a binary classification problem, consisting in deciding if a user is influential or not. To evaluate the classifier performance, Ramírez et al. [45] used the* F*-score, based on the Precision and Recall processed for each class, which is typical in classification tasks. The* F*-score is calculated as follows:

8 $$\begin{aligned} F = 2 \times \frac{ \left( P \times R \right) }{P + R} \end{aligned}$$

*P* and *R* are the Precision and Recall. This measure gives an overview of the system performance.

In our method, the influence degrees are presented by 8 classes (going from V.Weak until E.Strong), so, to be able to compare our approach with the real-world influence, we choose a threshold of influence degree; in other words, we consider that influence degrees under V.Strong are non influentials, and V.Strong and more are considered as influential.

#### Results and discussion

When we applied our method on the Replab data set, we found that the method was able to detect 92 among 144 influential users. Table 15 shows the *F*-measure results comparison between our method and some existing studies. Our method based on belief fusion has reached 0.584, and the measure is slightly lower than measures obtained form the state-of-the-art methods. This is due to many reasons: first, 36 % of the *tweets* were deleted from *Twitter*, thus, information about these *tweets* is no longer available, and this may bias the results. The positive aspects of our results must be modulated by the fact that users having the majority or all their *tweets* deleted have been assigned to the class $\Omega$, which means that our system is able to express its ignorance about the influence degree of users on which it has not enough information. Another reason for which the *F*-measure of our method is lower than the others, is that we do not consider the studied domain, and our method detects influential users regardless the studied domain; hence, many of the detected influential users by our method were not considered as influential in the Replab data set. When we studied these accounts, we found that these accounts seem to be influential in real life, but the reason why they were not labeled as influential in the Replab data set is that these accounts are not influential in the studied domains (automotive and banking).

**Table 15:** Table of* F*-score results comparison

	*F*-score
Cossu et al. [19]	0.792
Cossu et al. [46]	0.781
Ramírez et al. [45]	0.694
Belief fusion	0.584

## Conclusion

In this paper, we proposed an influence assessment approach for the *Twitter* social network. This approach addresses limitations of existing systems such as lack of relations combination and uncertainty ignorance on the given measures. In our work, we proposed an influence graph allowing us to observe different relations in the network, and we considered several relations and interactions: *retweet*, *mention*, *reply*, *retweet of replies*, and *retweet of mentions*. Based on the belief functions theory, we established a general influence measure for a given user by information fusion of the different relations. The proposed approach is flexible and can take into account different interaction patterns in the influence graph, and the influence measure considers influence exercised on indirect nodes (e.g., a user may *retweet* another’s *tweet* indirectly through an intermediate user).

We experimented our approach on real data gathered from *Twitter* in the context of the project TEE 2014 and the context of the Replab Challenge 2014. The experiments show that relations combination under uncertainty leads to a quite interesting results. Interesting perspectives emerge to further strengthen the proposed approach. The method for users ranking will be improved. Moreover, we will study the content of the *tweets* to study whether the exercised influence is positive or negative. Besides, we plan to apply the proposed approach on other measures that requires information fusion such as users credibility and *Twitter* styles categorization. And finally, we will consider more complex interaction patterns in the method, such as *hashtags* on a multi-relational graph.

## Footnotes

1
https://www.github.com/kerzol/Influence-assessment-in-twitter.

2
https://www.github.com/SNFreezer.

3
http://www.clef2014.clef-initiative.eu/.

4
http://www.llorenteycuenca.com/.

5
http://www.nlp.uned.es/replab2014/.

6
https://www.github.com/twitter/twurl.
